# The Investigation of Mycotoxins and Enterobacteriaceae of Cereal-Based Baby Foods Marketed in Turkey

**DOI:** 10.3390/foods10123040

**Published:** 2021-12-07

**Authors:** Buket Er Demirhan, Burak Demirhan

**Affiliations:** Department of Pharmaceutical Basic Sciences, Faculty of Pharmacy, Gazi University, Ankara 06330, Turkey; bdemirhan@gazi.edu.tr

**Keywords:** baby food, mycotoxins, microbiological analysis, LC-MS/MS

## Abstract

In this study, a total of 85 cereal-based baby foods with or without milk (four different brands; A, B, C, and D) collected from Ankara local markets, Turkey were analyzed for mycotoxins, total aerobic mesophilic bacteria (TAMB), and Enterobacteriaceae contamination. Baby foods were analyzed for 12 toxicological important mycotoxins such as aflatoxin B1, B2, G1, and G2; fumonisin B1 and B2; ochratoxin A; sterigmatocystin (STE); deoxynivalenol (DON); zearalenone (ZON); and T-2 toxin and HT-2 toxin by LC-MS/MS multi-mycotoxin method. In addition to these mycotoxins, the presence of aflatoxin M1 (AFM1) was investigated in baby foods containing milk. The classical culture method was used for microbiological analysis. Consequently, at least one mycotoxin was detected in 69.41% of the total samples. The most frequently detected mycotoxins were STE (34.12%) and HT-2 (34.12%). However, AFM1 was not detected in any of the baby foods containing milk. Also, TAMB and Enterobacteriaceae were isolated from 30.59% and 10.59% of samples, respectively. As a result, it was determined that the mycotoxin levels in the analyzed samples were in accordance with the mycotoxin levels specified in the Turkish Food Codex.

## 1. Introduction

Foodborne diseases resulting from microbiological contaminations are an important global public health problem. These diseases are very important to governments, industry, and consumers in the prevention of diseases and protection of human health [1]. Food safety is an important issue that directly affects public health and is the responsibility of public authorities and the food industry. Food safety risks, especially for which microbiological contamination is responsible, are a serious concern, causing health problems mainly in children under the age of 5 [2]. Infections are usually caused by the ingestion of potential pathogens with food. Even if heat treatments are applied, it is stated that care should be taken in the preparation of baby foods, as baby foods may contain live microorganisms [3]. Powdered baby food is not a sterile product, it is contaminated with pathogenic microorganisms and can cause food poisoning and other serious diseases in infants [4]. The major problem of baby foods production is microbiological contamination [5]. Cereals are one of the first solid foods that babies consume in their diets, but they also constitute an important source of nutrients [6,7]. Powdered baby foods are considered a food that is rehydrated by the consumer before use. The microbiological stability of powder baby foods is achieved through the dehydration process applied to the products as well as packaging that provides an effective barrier against contact with moisture and oxygen. Although the manufacturer of baby food uses some processes to reduce the microbial load in the product, baby food is not a completely sterile product and therefore may have low levels of microorganisms even when produced under controlled conditions [8]. The formulation of baby food may contain several components such as cereals, fruits, dairy products, and nuts. Inadequate food processing conditions cause contamination of baby food with microorganisms [9]. The total number of aerobic mesophilic bacteria is used to indicate general sanitation, microbiological quality, and deterioration in food matrices such as cooked ready-to-eat foods, pasteurized milk, and spices. Enterobacteriaceae are used to determine fecal contamination and the effectiveness of sanitation programs in food matrices such as powdered baby food and cooked ready-to-eat foods [8].

Mycotoxins are secondary metabolites produced by different molds, such as Aspergillus, Penicillium, and Fusarium genera that can grow on food under certain humidity and temperature conditions [10,11]. Many of the mycotoxins are relatively stable throughout food processing and can survive intact in final products [12,13]. Nowadays, over 300 mycotoxins are known [14]. Aflatoxins (AFs), ochratoxin A (OTA), zearalenone (ZEN), deoxynivalenol (DON), nivalenol (NIV), T-2, HT-2, patulin (PAT), and fumonisins (FBs) are the most common mycotoxins in foods [11]. Humans are exposed to mycotoxins, albeit at residual levels, through food and products derived from animals fed with contaminated feed [15]. Among all the mycotoxins, aflatoxin is the strongest and most toxic carcinogen [14]. They can occur in cereals, nuts, cocoa, dried fruit, wine, spices, red pepper products [16,17,18].

AFM1 is a hydroxylated metabolite of AFB1 and can be found in milk and dairy products. Due to their heat resistance, AFM1 cannot be degraded or destroyed by heat treatment procedures applied to food. Therefore, AFM1 residues in milk and dairy products are recognized as an important public health problem [19]. Another important mycotoxin is OTA, which has high toxicity and causes serious health problems [20]. OTA is stated to be a nephrotoxic factor [21]. It can be found in many food products including cereals, cereal products, coffee, cocoa beans, grapes, grape juice, beer, wine, spices, soy, nuts, milk products, and meat [18,22]. Fumonisins are mycotoxins produced by at least 11 Fusarium fungal species, including the maize pathogens *Fusarium verticillioides* and *Fusarium proliferatum*. As a result of epidemiological studies, it is stated that there is a relationship between exposure to fumonisin B1 (FB1) and esophageal cancer [13]. T-2 and HT-2 toxins are among Type A trichothecenes, while DON is a Type B trichothecene. Infants and young children were considered the highest risk group when they consumed large amounts of cereal-based foods relative to their body weight [23]. The health effects of these mycotoxins and other trichothecenes generally occur through the inhibition of protein, DNA, and RNA synthesis [21]. The main toxic effects of DON can be stated as irregularities in the immune and reproductive systems and growth disorders caused by anorexia [10]. When children consume heavily DON-contaminated food, vomiting could be seen within hours [13]. ZEN is a mycotoxin found in common foodstuffs and cereals in the world, produced by Fusarium species [24]. ZEN affects the reproductive system in children, affecting the structure and function of the reproductive organ, causing hyperestrogenism. ZENs are mycotoxins that affect the growth and development of children and may cause precocious puberty in children exposed to these compounds [13].

The presence of contaminants such as mycotoxins in the diet and especially in the foods of sensitive individuals such as infants is a major health problem [25]. In order to evaluate dietary exposure to mycotoxins in infants, it appears crucial to monitor their exposure through infant formula, follow-on formula, cereal-based foods, and baby foods for infants and young children [18]. Children are three times more sensitive to the toxic effects of mycotoxins than adults because children have lower body mass, higher metabolic rates, and do not yet have a mature detoxification system compared to adults [11]. Mycotoxins are important contaminants due to the cost of treatment of diseases caused by mycotoxins, as well as the economic damage they cause in international trade [21].

The aim of this study was to determine the presence of Enterobacteriaceae and total aerobic mesophilic bacteria (TAMB) and mycotoxin levels in baby food samples sold in Ankara local markets and to evaluate whether the levels were within the limits specified in the Turkish Food Codex (TFC).

## 2. Materials and Methods

### 2.1. Samples Collection

A total of 85 cereal-based baby food samples (with or without milk) from four different brands, A (*n* = 10), B (*n* = 24), C (*n* = 20), and D (*n* = 31), were collected from April to July 2021 in different local markets in Ankara, Turkey. Samples of A and B brands and 10 samples of C brand contain cereals with milk. Powdered baby food samples containing cereals include rice, oats, wheat semolina, barley, rye, millet, and wheat flours. Each of the samples brought to the laboratory for analysis had different batch numbers. Baby foods were kept in a dark and dry place at room temperature until analysis. These baby food brands are also sold almost all over Turkey.

### 2.2. Sample Preparation 

Sample preparations were done according to the instructions of the LC-MS/MS Jasem analysis kit (SEM, İstanbul, Turkey) [26]. For multi-mycotoxin analysis in cereal-based baby foods, 2.0 g of the homogenized baby food samples was transferred to 50 mL centrifuge tubes. Ten milliliters of reagent 1 (JSM FO 9704) was added to the sample and shaken for 15 min using a multi-shaker. Subsequently, this was centrifuged at 3000 rpm for 5 min at room temperature. The clear supernatant was filtered through a 0.45-micron nylon filter into an HPLC vial and injected directly into the LC-MS/MS instrument. In addition to the analysis of 12 mycotoxins in the cereal samples with milk, AFM1 analysis was also performed. In order to determine the AFM1 contamination of the cereal with milk samples, 1 g of each sample was diluted with 10 mL distilled water and mixed well. One milliliter of this solution was taken into a glass centrifuge tube. Then, 4 mL reagent 1(JSM FO 1503) was added and mixed for 15 s; and then centrifuged for four minutes at 3000 rpm. After centrifugation, the supernatant was filtered through a 0.45-micron nylon filter into an HPLC vial and directly applied on the LC-MS/MS.

### 2.3. LC–MS/MS Procedure 

The samples were screened for multi-mycotoxin (aflatoxin B1 (AFB1), AFB2, AFG1, and AFG2; fumonisin B1 (FB1), FB2; ochratoxin A (OTA); sterigmatocystin (STE); deoxynivalenol (DON); zearalenone (ZON); T-2 toxin and HT-2 toxin contents) with a commercial multi-mycotoxin LC-MS/MS Jasem kit (SEM, İstanbul, Turkey) [26]. For AFM1 analysis of cereal with milk samples, AFM1 LC-MS/MS Jasem kit (SEM, İstanbul, Turkey) was used. The assay was performed according to the guidelines of the manufacturers. Jasem HPLC solvents and calibrator solutions were used. All mycotoxins were analyzed by using an Agilent LC 1290 combined with 6470 triple quad mass spectrometer (LC-MS/MS) and Electrospray ionization (ESI) (Agilent Technologies, Santa Clara, CA, USA), equipped with a multi-mycotoxin analytical column (JSM-FO-9775) and AFM1 analytical column (JSM-FO-1575). For multi-mycotoxin, the injection volume was set to 10 µL and the flow rate 0.5 mL/min. The injection volume was set to 5 µL and the flow rate was 0.8 mL/min in AFM1 analysis in samples. In the analysis of all mycotoxins, column furnace temperature was set to 35 °C. For data acquisition, method creation, and qualitative and quantitative analysis, a MassHunter 647 Workstation version 10.1 (Agilent Technologies, Santa Clara, CA, USA) was used. Multi-mycotoxin and AFM1 levels in samples were expressed as parts per billion (ppb) and parts per trillion (ppt), respectively.

### 2.4. Estimation of Dietary Intake of Mycotoxins

Estimation of dietary mycotoxin exposure of infants was made based on the estimated dietary intake of mycotoxins from baby foods, based on the 6–12 months of infants (8.8 kg) and 1–3 years of toddlers mean body weight (12 kg), average baby food consumption of 0.28 kg/day, and average mycotoxin levels [27,28].

### 2.5. Bacteriological Analysis

The classical culture method was used for microbiological analysis of baby foods. For TAMB analysis, representative 10 g portions of baby food samples were aseptically weighed and homogenized with 90 mL of maximum recovery diluent (MRD, Merck 1.12535, Darmstadt, Germany) using a Stomacher lab-blender (Bagmixer 400, Saint-Nom-la-Bretèche, France). Twenty-five (25) grams of baby food samples and 225 mL of MRD were used in Enterobacteriaceae analysis. A threefold serial dilution was made using a 1 mL aliquot from the stock solution. Three serial dilutions were plated in duplicates onto plates. The violet red bile dextrose agar (VRBD, Merck 1.10275, Darmstadt, Germany) and Plate Count Agar (PCA, Merck 1.05463, Darmstadt, Germany) were used for enumeration of Enterobacteriaceae and TAMB, respectively. These plates were incubated at 37 °C for 24 h. At the end of incubation, total viable counts were expressed in colony forming units per gram of fresh weight sample (CFU/g) [29]. 

### 2.6. Statistics 

Descriptive analysis of multi-mycotoxin data obtained from four different brands of baby foods was carried out with SPSS 16 [30].

## 3. Results and Discussion

**Figure 1:**
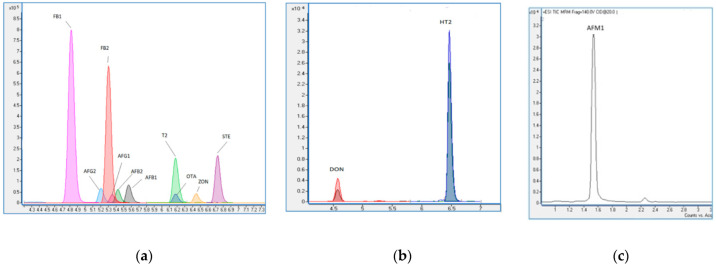
The LC-MS/MS chromatograms (TIC mode) of mycotoxins: (**a**) aflatoxin B1 (AFB1), aflatoxin B2 (AFB2), aflatoxin G1 (AFG1), aflatoxin G2 (AFG2), fumonisin B1 (FB1), fumonisin B2 (FB2), ochratoxin A (OTA), sterigmatocystin (STE), zearalenone (ZON), and T-2 toxin (T2); (**b**) deoxynivalenol (DON) and HT-2 toxin (HT2); and (**c**) aflatoxin M1 (AFM1).

A total of 85 baby food samples (four different brands; A, B, C, and D) were analyzed for the mycotoxin (AFB1, AFB2, AFG1, and AFG2; FB1, FB2; OTA; STE; DON; ZON; T-2 toxin; HT-2 toxin; and AFM1 contents) by LC-MS/MS and total aerobic mesophilic bacteria (TAMB) and Enterobacteriaceae contamination by classical methods. Within the last few years, it has been stated that the LC-MS/MS method is a universal approach for mycotoxin analysis in foods. This method has a high selectivity [14]. The LC-MS/MS chromatograms (TIC mode) of mycotoxins are shown in Figure 1. Validation parameters such as correlation coefficient (R2), the limit of detection (LOD), the limit of quantification (LOQ), relative standard deviation (RSD), and recovery were assessed (Table 1).

The levels of AFB1, AFB2, FB1, FB2, OTA, STE, DON, and HT-2 in all baby food samples were determined. However, AFG1, AFG2, ZON, and T-2 were not detected in all the samples. The AFM1 was investigated in only cereal with milk samples and not detected in the samples. At least one mycotoxin was detected in 69.41% of the total samples. The most frequently detected mycotoxins were STE (34.12%) and HT-2 (34.12%). The concerning values are shown in Table 2. Due to the vulnerability and exposure of infants and young children to mycotoxins in food, it is essential for public health to estimate dietary intake for mycotoxins, taking into account the maximum allowable mycotoxin levels in food. Since the data obtained in the calculation of estimated mycotoxin exposure for 6–12 months of infants and 1–3 years of toddlers are well below the tolerable daily intake (TDI) of the mycotoxin levels determined by the FAO/WHO expert JECFA committees and EFSA, it can be said that it may not pose a risk in terms of health. Although the estimated mycotoxin exposure is lower than the TDI values, monitoring the mycotoxin content in baby foods is very important for the protection of public health and ensure the food safety. Similar to our study, Ishikawa et al. [31] and Oueslati et al. [32] stated that there is no risk in terms of health in infants in line with the data obtained as a result of estimated mycotoxin exposure from infant foods. However, contrary to our study, Rodríguez-Carrasco et al. [33] and Adetunji et al. [34] stated that infants are at risk for health as a result of their study.

STE mycotoxin was detected in the samples of brand A, and the mean ± standard error (SE) value was found to be 0.084 ± 0.031 μg/kg. The mean and standard error values of AFB1, FB1, OTA, STE, and HT-2 mycotoxins in brand B were 0.036 ± 0.008 μg/kg, 4.70 ± 1.03 μg/kg, 0.07 ± 0.02 μg/kg, 0.07 ± 0.005 μg/kg, 0.07 ± 0.03 μg/kg, and 1.88 ± 0.07 μg/kg, respectively. In brand B, AFB2 toxin was detected as 0.03 μg/kg in only one sample and DON mycotoxin was not detected. The mean and standard error values of STE and HT-2 toxins in brand C were found to be 0.02 ± 0.003 μg/kg and 2.15 ± 0.22 μg/kg, respectively. AFB1, AFB2, FB1, FB2, OTA, and DON mycotoxins were not found in brand C. In the D brand, mean and standard error values of STE, DON, and HT-2 toxins were found to be 0.11 ± 0.02 μg/kg, 17.82 ± 2.85 μg/kg, and 1.64 ± 0.25 μg/kg, respectively. In brand D, AFB2 was detected as 0.06 μg/kg in only one sample (Table 3). In order to protect public health and ensure food safety, maximum levels for mycotoxins in some foods have been determined in many countries. The results of the analyses were evaluated within the Turkish Food Codex (TFC) values. In the TFC, the maximum levels (MLs) of AFB1, OTA, DON, and ZON in infant and child complementary foods were set as 0.10 µg/kg, 0.5 µg/kg, 200 µg/kg, and 20 µg/kg, respectively. In addition, the levels of ZON and fumonisin (B1 + B2) in processed corn-based infant and child complementary foods are specified as 20 µg/kg and 200 µg/kg, respectively. Regulation for MLs of AFB1, OTA, DON, ZON, and fumonisin (B1 + B2) in infant and child complementary foods in Turkey was prepared in parallel with the European Union (EU) Regulation [35]. It was found that the presence and amount of DON and FB1 differed according to the cereal type of infant foods. It was found that the amount of DON was high in baby foods containing wheat and rice, and FB1 was high in baby foods with high corn and millet content.

The scarcity of similar studies with multi-mycotoxin detection in baby foods in Turkey shows the importance of this study. To the best of our knowledge, this is the first study to investigate the presence of 12 mycotoxins in baby foods in Turkey. Commercial baby foods included in this study are distributed for sale almost all over Turkey. For this reason, the results of our research are also important in terms of giving information about the multi-mycotoxin contamination of these baby foods in Turkey. 

There are a few studies conducted in Turkey on the determination of some mycotoxins levels in infant formulas and baby foods. These studies are largely based on AFs and OTA. Baydar et al. [36] investigated the levels of AFB1, AFM1, and OTA in 63 infant formulas, follow-on formulas, and baby foods. They found that AFB1, AFM1, and OTA were 87%, 36.5%, and 40% of the samples, respectively. Er et al. [37] examined 50 follow-on milk and 34 infant formulas in Turkey (Ankara) for AFM1 contamination. AFM1 was detected in 32 (38.1%) of the samples. In another study, Hampikyan et al. [38] determined OTA in baby foods in İstanbul. They found OTA in 52 out of 150 (34.7%) samples. Kabak [39] examined 62 samples of baby formula for the AFM1 and OTA in Turkey. He found AFM1 and OTA in 5 (8%) and 12 (19.4%) samples, respectively. 

There are some studies on the contamination of multi-mycotoxins in baby food products in several countries of the world. Lombaert et al. [40] investigated DON, nivalenol, HT-2 toxin, ZON, OTA, FB1, and FB2, and five ergot alkaloids in 363 cereal-based infant foods they collected from markets in Canada. It was stated that DON, ZON, fumonisins, and OTA were detected in total samples at a rate of 63.3%, 32.6%, 29.6%, and 26.1%, respectively. Alvito et al. [25] investigated the amounts of AFB1, AFM1, and OTA in baby foods in Portugal. They stated that in 15 out of 27 samples, the levels of AFM1 (4 positive samples), AFB1 (1 positive sample), and OTA (10 positive samples) were found to be 0.017–0.041 μg/kg, 0.009 μg/kg, and 0.034–0.212 μg/kg, respectively. Kalantari et al. [41] analyzed AFB1, B2, G1, and G2 contamination in their study on infant food additives in Iran and stated that 4 of 29 samples were contaminated with AFB1 and AFB2 at doses lower than 2 ppb. Rubert et al. [42] investigated 21 mycotoxins in commercial infant formulas. They reported that mycotoxin levels in 35 commercial baby foods did not exceed the maximum values specified in the European Union directives. Assunção et al. [43] stated that they found 1 AFB2, 2 AFG1, 8 AFM1, 10 OTA, 7 FB1, 6 ZON, and 4 DON toxins in 20 infant cereal foods in their study on multi-mycotoxin exposure of children in Portugal. Juan et al. [17] investigated the presence of 23 different mycotoxins in 75 commercial infant foods in Italy. They stated that 31% of cereal-based infant foods contain mycotoxins and OTA, DON, and HT-2 levels are 20%, 21%, and 3%, respectively. Al-Taher et al. [44] reported that the number of positive samples containing DON, AFB1, AFB2, AFG2, OTA, ZEN, FB1, FB2, HT-2, and T-2 toxins in 64 infant foods obtained from US markets was 42, 3, 14, 9, 19, 13, 1, 5, 6, and 18, respectively. They also stated that they did not detect AFG1 toxin from infant foods.

In our study, TAMB was found in 30.59% of baby food samples (26/85), and Enterobacteriaceae was found in 10.59% of baby food samples (9/85). The results of Enterobacteriaceae and TAMB analysis of 85 baby food samples are summarized in Table 4. In the analysis of total aerobic mesophilic bacteria, no growth was observed in brand A, and the TAMB number was less than 10^2^ CFU/g in brand A. TAMB growth was observed in almost all samples of brands B. In D and C brands, TAMB was grown in two and one samples, respectively. Enterobacteriaceae growth was detected in one, four, two, and two samples of brands A, B, C, and D, respectively.

Generally, the number of TAMB in foods above 6 log CFU/g indicates that deterioration has started. It is assumed that the number of TAMB obtained in our study does not pose a risk in terms of food safety, considering that baby foods are not sterile. Since the Turkish Food Codex microbiological criteria regulation, the Enterobacteriaceae limit should be less than 1 log CFU/g in infant and young child supplementary foods (including special medical purpose diet foods), the mean Enterobacteriaceae count of the positive samples is 3.14 log CFU/g, and these mean level are above the TFC value. Enterobacteriaceae is very important in ensuring food safety and protecting public health, as it is an indicator in determining the fecal contamination and efficiency of sanitation. There are some studies on the microbial contamination in baby food products in Turkey and several countries of the world. Sezer et al. [45] investigated the microbiological quality of infant milk and follow-up products. For this purpose, they stated that TAMB growth was detected in 29 of 50 samples. Heperkan et al. [46] found the number of mesophilic aerobic bacteria in the powdered infant formula and follow-up formula as 1.71 and 1.95 log CFU/g, respectively. Kim et al. [47] investigated microbial contamination in foods consumed by infants and babies in South Korea, and it was reported that they detected the Enterobacteriaceae family in 14 of 75 (18.7%) powdered infant formulas in their study. It is also stated that they detected aerobic plate counts in 96 of 100 cereal-based follow-up formulas. Sani et al. [48] investigated the microbiological quality of powdered infant formulas, follow-up formulas, and infant formulas as a result of rehydration and found aerobic plate counts in 29 of 90 samples. Sadek et al. [49] stated that they found the mean total bacterial count in all 20 rice and wheat-based infant foods as 2.923 and 2.856 log CFU/g, respectively.

## 4. Conclusions

Baby foods can be frequently contaminated with various kinds of mycotoxins. In this study, AFB1, AFB2, FB1, FB2, OTA, STE, DON, and HT-2 were found in 12.94, 3.53, 12.94, 10.59, 2.35, 34.12, 14.12, and 34.12% of analyzed baby food samples, respectively. The levels of mycotoxins in positive samples were found to be below the specified values in TFC. Other mycotoxins including AFG1, AFG2, AFB2, and T-2 were not detected in any of the samples. In addition, the AFM1 was investigated in only cereal with milk samples and not detected in these samples. Contamination rates of TAMB and Enterobacteriaceae were found in 30.59% and 10.59% of all baby food samples, respectively. Finally, mycotoxins and microorganisms cause serious risks to food safety and human health, especially in infants and young children, while also causing economic losses to the food industry. From an economic point of view, it is important to save mycotoxin-contaminated food products and prevent the health risks associated with mycotoxins. Maize is one of the grains richest in aflatoxin formation, and even pre-harvest crops can be exposed to high levels of aflatoxin-producing fungus. Other grain crops such as wheat, barley, oats, and sorghum are not particularly susceptible to heavy pre-harvest aflatoxin contamination. It is stated that the mycotoxin contamination of small grains is lower compared to large grains, and some small grains do not contain any mycotoxins. Therefore, it is recommended to grow smaller grains to reduce mycotoxin formation. Another option may be to replace grains that are highly susceptible to mycotoxin contamination, such as maize, with grains that are less susceptible to mycotoxin contamination, such as sorghum or millet. As a result, consumers, especially children, may be less exposed to mycotoxins in their diets. Sorting grains before storage, improving harvesting methods, and peeling grains can be considered as ways to reduce mycotoxin contamination. At the same time, it is important to control and monitor with analytical methods from the field to the fork of foods. 

## Figures and Tables

**Table 1:** Validation parameters of applied LC-MS/MS method.

Mycotoxins	R^2^	LOD	LOQ	RSD (%)	% REC.
AFB1	0.997	0.01	0.02	7.27	98.11
AFB2	0.998	0.02	0.07	4.9	102
AFG1	0.998	0.04	0.12	3.41	102.34
AFG2	0.997	0.04	0.15	6.71	96.3
AFM1	0.996	0.03	0.01	4.18	106
FB1	0.998	0.58	1.93	3.27	82.53
FB2	0.998	0.24	0.80	2.96	92.97
OTA	0.997	0.01	0.03	3.65	91.17
STE	0.999	0.02	0.07	3.53	100.27
DON	0.997	2.40	8.01	5.52	108.67
ZON	0.997	0.13	0.45	3.93	107.61
HT2	0.997	0.40	1.35	8.00	97.94
T2	0.998	0.15	0.49	4.78	96.47

AFB1: aflatoxin B1, AFB2: aflatoxin B2, AFG1: aflatoxin G1, AFG2: aflatoxin G2, FB1: fumonisin B1, FB2: fumonisin B2, OTA: ochratoxin A, STE: sterigmatocystin, DON: deoxynivalenol, ZON: zearalenone, HT2: HT-2 toxin and T2: T-2 toxin.

**Table 2:** The levels of mycotoxins in all baby foods.

Mycotoxins	Positive Samples (%)	Concentration (μg/kg)		Estimation of Daily Exposure of Aged 6–12 Months (μg/kg/bw)	Estimation of Daily Exposure of Aged 1–3 Years (μg/kg/bw)
		Minimum and Maximum	Mean ± S.E		
AFB1	12.94	0.01–0.08	0.036 ± 0.008	0.001	0.0008
AFB2	3.53	0.03–0.06	0.05 ± 0.01	0.002	0.001
AFG1	nd	nd	nd	nd	nd
AFG2	nd	nd	nd	nd	nd
AFM1 *	nd	nd	nd	nd	nd
FB1	12.94	2.01–14.09	4.70 ± 1.025	0.15	0.11
FB2	10.59	0.04–0.20	0.071 ± 0.017	0.002	0.002
OTA	2.35	0.06–0.07	0.065 ± 0.005	0.002	0.002
STE	34.12	0.01–0.50	0.063 ± 0.018	0.002	0.002
DON	14.12	6.32–37.52	17.82 ± 2.85	0.57	0.42
ZON	nd	nd	nd	nd	nd
T2	nd	nd	nd	nd	nd
HT2	34.12	0.02–3.31	1.76 ± 0.15	0.06	0.04

nd: not detected, *: Value for 44 samples containing milk, AFB1: aflatoxin B1, AFB2: aflatoxin B2, AFG1: aflatoxin G1, AFG2: aflatoxin G2, FB1: fumonisin B1, FB2: fumonisin B2, OTA: ochratoxin A, STE: sterigmatocystin, DON: deox-ynivalenol, ZON: zearalenone, T2: T-2 toxin and HT2: HT-2 toxin.

**Table 3:** Minimum, maximum, and total mycotoxin values in positive samples of brands.

		Brands			
		A	B	C	D
AFB1	**N**	10	24	20	31
	**Mean ± S.E (μg/kg)**	nd	0.036 ± 0.008	nd	nd
	**Min (μg/kg)**	nd	0.01	nd	nd
	**Max (μg/kg)**	nd	0.08	nd	nd
AFB2	**N**	10	24	20	31
	**Mean ± S.E (μg/kg)**	nd	0.03	nd	0.06
	**Min (μg/kg)**	nd	nd	nd	0.06
	**Max (μg/kg)**	nd	0.03	nd	0.06
FB1	**N**	10	24	20	31
	**Mean ± S.E (μg/kg)**	nd	4.70 ± 1.03	nd	nd
	**Min (μg/kg)**	nd	2.01	nd	nd
	**Max (μg/kg)**	nd	14.09	nd	nd
FB2	**N**	10	24	20	31
	**Mean ± S.E (μg/kg)**	nd	0.07 ± 0.02	nd	nd
	**Min (μg/kg)**	nd	0.04	nd	nd
	**Max (μg/kg)**	nd	0.20	nd	nd
OTA	**N**	10	24	20	31
	**Mean ± S.E (μg/kg)**	nd	0.07 ± 0.005	nd	nd
	**Min (μg/kg)**	nd	0.06	nd	nd
	**Max (μg/kg)**	nd	0.07	nd	nd
STE	**N**	10	24	20	31
	**Mean ± S.E (μg/kg)**	0.084 ± 0.031	0.07 ± 0.03	0.02 ± 0.003	0.11 ± 0.02
	**Min (μg/kg)**	0.01	0.01	0.01	0.08
	**Max (μg/kg)**	0.16	0.50	0.03	0.13
DON	**N**	10	24	20	31
	**Mean ± S.E (μg/kg)**	nd	nd	nd	17.82 ± 2.85
	**Min (μg/kg)**	nd	nd	nd	6.32
	**Max (μg/kg)**	nd	nd	nd	37.52
HT2	**N**	10	24	20	31
	**Mean ± S.E (μg/kg)**	nd	1.88 ± 0.07	2.15 ± 0.22	1.64 ± 0.25
	**Min (μg/kg)**	nd	1.53	1.93	0.02
	**Max (μg/kg)**	nd	2.19	2.37	3.31

nd: not detected, AFB1: aflatoxin B1, AFB2: aflatoxin B2, FB1: fumonisin B1, FB2: fumonisin B2, OTA: ochratoxin A, STE: sterigmatocystin, DON: deox-ynivalenol and HT2: HT-2 toxin.

**Table 4:** Contamination of Enterobacteriaceae and TAMB in baby foods.

	Positive Samples (%)	Number of Positive Samples	Mean ± SD (log CFU/g)
Enterobacteriaceae	10.59	9	3.14 ± 0.38
TAMB	30.59	26	3.09 ± 0.50

TAMB: Total aerobic mesophilic bacteria.
